# Identification of breeding objectives for Begait goat in western Tigray, North Ethiopia

**DOI:** 10.1007/s11250-018-1640-5

**Published:** 2018-06-21

**Authors:** Hagos Abraham, Solomon Gizaw, Mengistu Urge

**Affiliations:** 1 Department of Animal Science, Bule Hora University, P.O. Box 144, Bule Hora, Ethiopia; 2 International Livestock Research Institute (ILRI), P.O. Box 5689, Addis Ababa, Ethiopia; 3 School of Animal and Range Sciences, Haramaya University, P.O. Box 138, Dire Dawa, Ethiopia

**Keywords:** Bio-economic model, Economic parameters, Own-flock ranking, Performance traits

## Abstract

A sound breeding objective is the basis for genetic improvement in overall economic merit of farm animals. Begait goat is one of the identified breeds in Ethiopia, which is a multipurpose breed as it serves as source of cash income and source of food (meat and milk). Despite its importance, no formal breeding objectives exist for Begait goat. The objective of the present study was to identify breeding objectives for the breed through two approaches: using own-flock ranking experiment and developing deterministic bio-economic models as a preliminary step towards designing sustainable breeding programs for the breed. In the own-flock ranking experiment, a total of 45 households were visited at their homesteads and were asked to select, with reasons, the first best, second best, third best, and the most inferior does from their own flock. Age, previous reproduction, and production information of the identified animals were inquired; live body weight and some linear body measurements were taken. The bio-economic model included performance traits (weights, daily weight gain, kidding interval, litter size, milk yield, kid mortality, pregnancy, and replacement rates) and economic (revenue and costs) parameters. It was observed that there was close agreement between the farmers’ ranking and bio-economic model results. In general, the results of the present study indicated that Begait goat owners could improve performance of their goats and profitability of their farms by selecting for 6-month weight, litter size, pre-weaning kid survival rate, and milk yield.

## Introduction

Ethiopia is home for an estimated of 30 million goats (CSA 2016), which are distributed in different agro-ecological zones and mainly raised in the lowlands. In the last 10 years, the goat population in the country increased more rapidly (134%) than the sheep (65%) and cattle (38%) populations (FAOSTAT 2016). Goat meat production in Ethiopia is also increased by 2% between 2005 and 2012 and it was expected to be doubled in 2016 because of increased domestic and export market demands for goat meat (Legese and Fadiga 2014).

In spite of the large goat population and increased goat meat demand in the country, the present level of productivity is low. For instance, during the year 2013, goat contributed only 11.0 and 1.4% of the national meat and milk production, respectively (FAOSTAT 2016). There are different factors that contribute for the low productivity. Among those reasons for this failure, lack of sustainable breeding programs/schemes is an important barrier.

Genetic improvement programs focus at a directional improvement in genetics of animals in coming generations such that they will produce the desired products more efficiently in the future economic, social and ecological production environment (Groen 2000). The development of the breeding programs involves a precise definition of breeding objectives. A breeding objective defines the direction in which the farmer would like to go towards satisfying his demand for specific products and services from the animals (Sölkner et al. 2008), which usually translates into increase of profit (Nandolo et al. 2016). Breeding objective traits have to be easy to measure, heritable, variable, and not too many. Moreover, those traits should be included in the aggregate genotype according to their economic importance (Philipsson et al. 2006).

Breeding objectives can be identified through participatory approaches (Nielsen and Amer 2007; Dana et al. 2010; Mirkena 2010; Duguma et al. 2011) and bio-economic models (Byrne et al. 2010; Gizaw et al. 2010; Gunia et al. 2012; Laske et al. 2012; Lopes et al. 2012). However, in most cases, it has been missing while designing the breeding programs (Sölkner et al. 1998). For this reason, the objective of the present study was to identify breeding objectives for Begait goat through participatory approach and by developing bio-economic models in western zone of Tigray region of Ethiopia, which is the home tract of the breed.

## Materials and methods

### Description of the study area

This research work was conducted in Kafta Humera district of western zone of Tigray National Regional State. The study area is situated 1372 km away from the administrative center of Addis Ababa city to northwest direction. Geographically, it is located in between the latitudes 13° 14′–14° 27′ N and longitudes 36° 27′–37° 32′ E. The study area comprises *kolla* (lowland) and *weinadega* (midland) agro-climatic zones with an altitude ranging from 560 to 1849 m above sea level. The annual precipitation in the lowlands and midlands is 448.8 and 1102.5 mm, respectively (EARO 2002) occurring between June and September. The mean minimum and maximum temperatures in the lowlands are 25 and 27.5 °C, respectively, while the corresponding values in the midlands are 20 and 25 °C. The hottest months are between April and June with temperatures rising up to 42 °C. Detailed descriptions of the study area are indicated in previous works (Abraham et al. 2017a).

### Identification of breeding objectives

In the present study, we identified breeding objectives for Begait goat using participatory approach (own-flock ranking experiment) and through developing deterministic bio-economic models that are adopted from Mirkena (2010) and Gizaw et al. (2010), respectively. The methodologies are explained in the subsequent sections.

### Using participatory approach: own-flock ranking experiment

The experiment was conducted during October 2016. Forty-five Begait goat owners were visited in the morning before animals were released for grazing. Family members of selected households were asked to identify, with reasons, their first best, second best, third best, and the most worst does. Some morphometric measurements were taken on the identified does. The measurements included body size measurements (body length and chest girth) and body weight. Besides, age and previous life history of each individual ranked doe were taken for reproduction (number of kids born, litter size, number of kidding, and number of kids weaned) and production (average daily milk yield) parameters.

### Data analysis

The proportions of traits preferred by owners were analyzed by using frequency procedure of the statistical software SAS (2008). Body weight, linear body measurements, and other traits from the life history were analyzed using general linear model procedure fitting rank as fixed effect. Age effect (categorical variable) on quantitative traits (continuous dependent variables) was tested through analysis of co-variance (SPSS for windows, release 16.0, SPSS 2007).

### Using bio-economic models

A bio-economic modeling in livestock production systems presents the opportunity for incorporating some elements of human decision-making and simulates the impact of such decisions by using mathematical relationships produced from both biological and economic parameters (Rewe and Kahi 2012). In this study, the four steps suggested by Gizaw et al. (2010) were followed in identifying the breeding objectives: (a) definition of the production and marketing systems, (b) identification of sources of revenue and expenses, (c) identification of breeding objective traits (determining biological traits that affect revenue and costs), and (d) derivation of economic value of each trait.

### Definition of the production and marketing systems

The characterization of the production and marketing systems was obtained from interviews with farmers using pre-tested structured questionnaire and focus group discussions (Abraham et al. 2017a). The production system was defined as subsistence type, where feeding was based on unimproved native pastures. Begait goats spend through grazing and browsing in communal grazing areas all the daytime. As far as marketing system is concerned, the farmers most likely sell males with good body condition at their early ages to generate direct cash to pay back credit and other expenses (health, school, farm input, cropland rent, and laborer), but inferior males are remained in the flock. Dry matter, energy, and protein requirements for maintenance, pregnancy, lactation, growth, and mating/buck were calculated. The NRC (1981) standards were followed.

### Sources of revenue and costs

Progeny structures for a village flock of 350 does were defined based on reproduction parameters for Begait goat to calculate revenues and costs (Table 1). Revenues and costs were calculated on per doe basis. Profit was calculated as the difference between revenues and costs per doe joined per year. Sources of revenues included only tangible (direct regular cash income from sale of 6-month-old male and female kids, yearling unfinished male and female kids, milk, culled does, and bucks) benefits. The prices used in the present study were the market prices of June 2017 in the study area, which were collected primarily through informal interview of key informants and personal observations. Costs included feed, veterinary, management, marketing, and fixed costs.

**Table 1:** Sources of revenues and costs used for defining breeding objectives of Begait goat

Sources of revenue	Unit	Value
Male kids (6 months old)	ETB/kg of live weight	95.43
Female kids (6 months old)	ETB/kg of live weight	89.47
Unfinished male kids (yearling)	ETB/kg of live weight	38.72
Unfinished female kids (yearling)	ETB/kg of live weight	34.25
Unfinished buck	ETB/kg of live weight	85.76
Unfinished doe	ETB/kg of live weight	45.57
Milk	ETB/kg	20.00
Sources of costs		
Feed cost		
Grass hay	ETB/kg	2.38
Sesame seed cake	ETB/kg	5.40
Veterinary and management cost		
De-worming	ETB/kg BW/year	4.00
Spray against external parasites	ETB/head/year	0.00
Vaccinations	ETB/head/year	9.00
Veterinary treatment and drugs	ETB/head/year	8.00
Management†	ETB/doe/year	0.88
Marketing†	ETB/doe/year	2.65
Fixed costs	ETB/head/year	34.32

†Management (herding and feeding) and marketing cost (personal expenses, marketing tax). *ETB* Ethiopian birr

### Identification of breeding objective traits

When considering developing a criterion for selection so as to maximize the profit in the progeny generation, the basis of selection of parents is an important issue. In the development of breeding objective/goal, various traits that are known to influence profitability of livestock farm as well as observable attributes like color, appearance, and beauty can be considered. In the present study, only economically relevant traits that directly influence profitability of the farm were considered. Biological traits that influence revenue and costs associated with products of the subsistence production system were 6-month weight, post-weaning average daily weight gain, mature weight, litter size, pre-weaning kid survival rate, kidding interval, and milk yield.

### Derivation of economic value

Bio-economic models relating the different breeding objective traits with the components of production and marketing in the subsistence Begait goat production system were constructed. Microsoft Excel spreadsheets were used to develop the models (Gizaw 2016). The developed bio-economic models included performance traits and economic parameters (Tables 1 and 2). The models were designed so that effects of genetic improvement in breeding objective traits could reveal changes in values of revenue and costs in the defined production system.

**Table 2:** Production parameters used for defining breeding objectives of Begait goat

Production parameters	Unit	Mean
Conception rate	%	90.00
Kidding interval	days	242.86
Litter size^**‡**^	kids	1.63
Survival (0–3 months), singles	%	88.00
Survival (0–3 months), multiples	%	85.00
Survival (3–6 months)	%	93.00
Survival (6–12 months)	%	96.00
Mortality (breeding does/bucks)	%	3.00
Doe weight	kg	35.50
Buck weight	kg	46.40
Kid weight (3 months, male, single-born)	kg	10.48
Kid weight (3 months, male, twin-born)	kg	9.54
Kid weight (3 months, female, single-born)	kg	9.50
Kid weight (3 months, female, twin-born)	kg	8.48
Kid ADG (3–6 months, male single-born)	kg/day	0.074
Kid ADG (3–6 months, male twin-born)	kg/day	0.070
Kid ADG (3–6 months, female single-born)	kg/day	0.067
Kid ADG (3–6 months, female twin-born)	kg/day	0.065
Kid ADG, male (6–12 months on grazing)	kg/day	0.063
Kid ADG, female (6–12 months on grazing)	kg/day	0.060
Milk yield (kg/doe/day, single kid)	kg	0.650
Milk yield (kg/doe/day, twin kid)	kg	0.690
Proportion of milk harvested for sale/consumption	%	50.00

^‡^Litter size at birth. *ADG* average daily weight gain

Marginal economic values for each trait were estimated as a change in profit resulting from an increase in one additive genetic standard deviation in the trait value due to selection. Additive genetic standard deviations (*σ*_a_) were calculated using the formula: *σ*_a_ = $\sqrt{h2\times \sigma 2p}$, where *h*^2^ is the heritability and σ^2^*p* is the phenotypic variance. As performance recording has not yet been carried out by farmers and monitoring data were not sufficient enough, heritability estimates were taken from national and international literature summarized by Jembere (2016). Phenotypic variances were calculated from data collected from the Begait goat monitoring study (Abraham et al. 2017b; Abraham et al. in press). The production parameters that are used for defining breeding objectives of Begait goat are presented in Table 2.

## Results and discussion

### Breeding objective using participatory approach

Frequencies of stated preferences were calculated for total respondents. Table 3 presents results of does’ preferred traits from own-flock ranking experiment. Farmers preferred many traits of a breeding doe, which reflect its importance and multi-functional nature in the study area. Previous research findings from Ethiopia (Gebreyesus et al. 2013; Abraham et al. 2017a) and elsewhere in the Africa continent (Jaitner et al. 2001; Bett et al. 2009; Kosgey et al. 2008) revealed that there are different traits preferences for breeding does. The obtained results indicated that body size, twinning ability (multiple-birth), milk yield, and pre-weaning kid survival rate were often listed as preferred traits of breeding does representing 21.25, 20.00, 18.13, and 11.25%, respectively, of the total proportions of traits mentioned. Other important traits of preferences were mothering ability (9.38%), drought resistant (5.00%), kid weight at birth (3.75%), and kid sex (3.75%). In general, the reasons mentioned for the selection of the best quality does were intended to reflect what traits of Begait goat were appreciated by owners.

**Table 3:** List and frequency of does’ traits preferences from own-flock ranking experiment

Traits	Frequency	Percent
Body size	34	21.25
Milk yield	29	18.13
Kid sex	6	3.75
Kid survival	18	11.25
Twining ability (multiple-birth)	32	20.00
Mothering ability	15	9.38
Kidding interval	7	4.38
Kid weight at birth	6	3.75
Drought resistant	8	5.00
Temperament	1	0.63
Beauty	1	0.63
Udder size	3	1.88
Sum	160	100.00

The mean (± SE) values for age (in years), some performance traits, and linear body measurements of the identified breeding does are summarized in Table 4. The results of the present study indicated that the does chosen as the best quality had higher mean values for all traits (age, body weight, chest girth, body length, number of kidding, milk yield, number of kids born, and weaned) under study compared with the other does ranked as the most inferior ones. For instance, the magnitude difference between the best quality and the most inferior does in live body weight, twining ability, number of kids weaned, and milk yield were 11.09 kg, 0.50 kids, 9.89 kids, and 0.36 kg per day, respectively. In line with the present findings, previous studies (Mirkena 2010; König et al. 2016; Nandolo et al. 2016) reported similar traits preferences of farmers for sheep and goats. Besides, the longer age of best does indicated that farmers keep them for long period for achieving their objectives.

**Table 4:** Means (± SE) of body weight and traits from the life history of the ranked does

Traits	*p*	Rank			
		1	2	3	Inferior
Age (year)	***	6.47 ± 0.18^a^	4.47 ± 0.13^b^	3.53 ± 0.09^c^	2.56 ± 0.07^d^
Body weight (kg)	***	36.38 ± 0.24^a^	33.14 ± 0.31^b^	29.26 ± 0.31^c^	25.29 ± 0.34^d^
Chest girth (cm)	***	77.36 ± 0.55^a^	73.30 ± 0.46^b^	70.99 ± 0.42^c^	67.68 ± 0.39^d^
Body length (cm)	***	73.96 ± 0.49^a^	70.20 ± 0.38^b^	67.72 ± 0.48^c^	64.40 ± 0.37^d^
Number of kidding	***	6.49 ± 0.21^a^	4.47 ± 0.15^b^	3.47 ± 0.10^c^	2.54 ± 0.09^d^
Twinning (kids/doe/kidding)	***	1.88 ± 0.02^a^	1.70 ± 0.02^b^	1.56 ± 0.07^c^	1.38 ± 0.03^d^
Kids born (number)	***	12.20 ± 0.42^a^	7.58 ± 0.26^b^	5.38 ± 0.14^c^	3.53 ± 0.14^d^
Kids weaned (number)	***	11.53 ± 0.40^a^	6.07 ± 0.23^b^	3.73 ± 0.14^c^	1.64 ± 0.09^d^
Daily milk yield (kg)	***	0.79 ± 0.02^a^	0.71 ± 0.01^b^	0.64 ± 0.01^c^	0.42 ± 0.01^d^

Means on the same row with different superscript letters are significantly different; ***p < 0.001

### Breeding objective using bio-economic model

Estimation of economic value for all traits was positive, except for mature body weight of doe. Positive economic values indicated that genetic improvement in the traits would result in a positive effect on profitability. However, mature body weight was the least economically important trait in the existing production system. The obtained negative economic value for mature body weight of doe was in agreement with the result reported by different researchers (Vatankhah 2010; Bett et al. 2011; Borzi et al. 2017). The traits with the highest economic value were obtained for litter size, 6-month weight, pre-weaning kid survival rate, and milk yield because they have direct relation to revenue or costs in the defined production system. Wolfová and Wolf (2013) demonstrated that economic values are specific to production systems and market contexts. The marginal economic values of the traits are given in Table 5.

**Table 5:** *Undiscounted marginal economic values per unit increase in genetic merit for each trait in the defined production system

Breeding objective traits	Economic values
6-month weight	121.79
Average daily gain (3–6 months)	97.58
Mature weight	− 63.97
Litter size	206.82
Pre-weaning kid survival (0–3 months)	118.97
Kidding interval	60.09
Daily milk yield	110.08

*ZPLAN requires undiscounted economic values for further analysis

A high correspondence was observed between farmers’ ranking of traits and the estimated economic values of traits. The correspondence between the two methods was evaluated for traits that were common for the two methods, namely body size/6-month weight, milk yield, kid survival, twining ability (multiple-birth), and kidding interval. The correlation between proportions of farmers ranking the traits as most preferred (first rank, see Table 3) and economic values of traits (Table 5) was 0.71. The correlation between the rankings of the traits by the two methods was 0.80. This shows that farmers consider the economic benefits from improving traits when ranking the traits although farmers do consider multiple objectives including risk aversion. Therefore, for increasing flock profit, more attention should be given to genetic improvement of traits with large economic value and highly preferred by owners including litter size, 6-month weight, pre-weaning kid survival rate, and milk yield.

## Conclusion

Breeding objectives were identified for Begait goat through participatory approach (own-flock ranking experiment) and by developing deterministic bio-economic models. Indeed, the use of defined breeding objectives by the goat owners is important in order to increase performance. Findings of the present study show that the traits desired by farmers in the own-flock ranking experiment were in close agreement to those traits identified using bio-economic models, predominantly for twining ability (multiple-birth), body size (6-month weight), pre-weaning kid survival rate, and milk yield.

Simultaneous use of farmers’ preferences and bio-economic modeling would help to evaluate the economic returns to invest in the breeding program, while accommodating farmers’ preferences as well as estimating traits weights for use in constructing selection index. It can be recommended from the present findings that a multi-trait selection program could be designed using selection index containing twinning ability, body size, pre-weaning kid survival rate, and milk yield.
